# Integration of the Confucian Culture on Cross-Cultural Conflict Management: The Role of the COVID-19 Pandemic

**DOI:** 10.3389/fpsyg.2021.694646

**Published:** 2021-06-10

**Authors:** Xiaoqin Liu, Wenzhong Zhu, Yanmei Liang

**Affiliations:** ^1^School of Foreign Languages and Cultures, Guangdong University of Finance, Guangzhou, China; ^2^School of Business, Guangdong University of Foreign Studies, Guangzhou, China

**Keywords:** the Belt and Road initiative, COVID-19 pandemic, confucian culture, cross-cultural conflict management, integration

## Abstract

Cross-culture conflict management is the major challenge for the Chinese enterprises going global along the Belt and Road Initiative. This study explores the feasibility of integrating the Confucian culture into cross-culture conflict management, and a special role is given to the COVID-19 pandemic. We combine the Confucian culture values and Hofstede’s cultural dimension theory and adopt the questionnaire survey methods on the Chinese multinational enterprises’ employees. The Cronbach’s Alpha method is also deployed to test the reliability and validity of the data. We find the significant integration of the Confucian culture into cross-culture conflict management. Furthermore, 16 sub-values of the Confucian culture are suggested to mitigate the cross-culture conflicts in multinational enterprises effectively. The findings imply that Chinese enterprises should consider new strategies to manage the cross-culture conflicts, especially during the COVID-19 pandemic.

## Introduction

“The Belt and Road Initiative” (hereinafter “BRI”), abbreviated from the twenty-first century Maritime Silk Road and Silk Road Economic Zone, is an initiative proposed by President Xi Jinping and was initially implemented in 2013, aiming to establish a new model of international relations featuring Win-Win cooperation and forge a community of shared future of humankind. “BRI” advocates a peaceful and amicable international environment and enhances economic development with cooperation with the rest of the world. As for business cooperation with companies worldwide, China seeks harmony without uniformity and fosters amity with the neighboring countries. “BRI” shows China’s commitment to mutual benefits with the countries and the pursuit of economic prosperity all over the world.

With the progression of “BRI,” Chinese enterprises are encouraged to go global and have actively carried on various business cooperation with the countries along “BRI.” However, with the prosperous development chances, Chinese enterprises have to face cross-culture conflicts due to different cultural environments such as multinational employees and differences in host country culture. Effectiveness in cross-culture conflicts is becoming the foremost challenge if Chinese enterprises want to achieve sustainable international business development. Furthermore, cross-culture conflict management’s success is also crucial for people in the host countries to properly interpret China’s “BRI” and then embrace it willingly.

Xi Jinping proposed that exchanges and mutual learning among civilizations should follow diversity, equality and inclusiveness. “Diversity” advocates building a world civilization composed of diverse cultures. “Equality” encourages equal exchanges and mutual learning between different cultures; “Inclusiveness” aims to achieve “coexistence of civilizations” through cultural exchanges and integration. Chinese enterprises consider these principles and are suggested to solve these cross-cultural conflicts harmoniously. Our study introduces Confucian culture into the management of cross-cultural conflicts. Especially the four core values, “He,” “Ren,” “Li,” and “Zhongyong” proposed by Tsai et al. (2011), are precisely discussed. In combination with Hofstede’s four dimensions, a theoretical research framework has been established to analyze how these Confucian culture values are integrated into the management of cross-culture conflicts for the Chinese enterprises going global. With the theoretical research framework’s guidance, an empirical study has been done among Chinese multinational enterprises’ cross-cultural employees.

The research findings put forward practical strategic suggestions on how the management takes advantage of the Confucian culture’s essence to mitigate the cross-culture conflicts. Furthermore, this research also enriches cross-cultural conflict management literature from the new perspective of Confucian culture integration. Lastly, this study contributes to enhancing the mutual understanding of different cultures in different countries along “BRI.” On the one hand, Chinese enterprises should advocate Confucian culture inside their companies and contribute Confucian culture wisdom to the global economy’s development. On the other hand, Chines enterprises take the role of an ambassador of friendship for exchanges between China and foreign countries.

The rest of the paper is organized as follows. Section “Literature Review” reviews the previous papers. Section “Methodology and Data” explains the data and the methodology. Section “Findings’ provides the findings. Section “Conclusion” concludes.

## Literature Review

Cross-cultural conflict management in multinational corporations refers to managing business organizations and diverse employees of different national or cultural backgrounds. The related studies have tried to understand the effective business management mode or methodology of cross-cultural conflicts in the global context of different cultural communities with inter or cross-cultural conditions. Several papers have focused on national culture variables and further tried to provide insights or solutions for the enterprises’ cross-cultural conflicts dilemma (Kogut and Singh, 1988; Jiang et al., 2016).

Cross-cultural management is an influential discipline, and several researchers have done studies on cross-cultural management issues. Hofstede (1980) conducted two-round questionnaire studies on IBM employees worldwide to understand the cultural differences and their impacts on values, behaviors, norms and institutions. Thus, the author proposed an analysis model of cultural dimensions, including power distance, uncertainty avoidance index, individualism vs. collectivism, and masculine vs. femininity. Later on, the author adopted other scholars’ ideas to expand the model to cover another two dimensions of long-term vs. short-term and indulgence vs. restraint. Besides Hofstede’s study, some other studies proposed that workplace cultural diversity was potentially beneficial, enhancing productivity and innovation. It could also intensify conflicts due to cultural clashes, such as the differences in values and working behaviors (Mannix and Neale, 2005).

This approach is also tested with the data for Chinese firms. Some Chinese researchers have focused on studying the multinational enterprises along “BRI,” bridging and adjusting modes of cross-cultural conflict management. In general, these researches attached importance to cross-cultural conflict management. They attempted to propose modes and adjusted communication approaches that effectively resolved cross-cultural conflict issues for multinationals. It is found that foreign-invested companies should start from reality and construct a new business operation model in the local markets by integrating different cultural elements to their new corporate cultural norms, thus resolving the inner cross-cultural conflicts faced by these multinational group companies. For instance, Chin (2015) argued that harmony played an important role in promoting organizational citizenship behaviors in Chinese organizations, thus managing cross-cultural conflicts effectively. Lu et al. (2019) studied social responsibility toward the employees and career development sustainability during manufacturing transformation in China. They proposed that Chinese firms should establish a sustainable culture in their business operation facing globalization. Liu et al. (2020) explored the interactive effects of national culture differences and conflict management approaches on international construction joint ventures’ performance.

To sum up, there are many valuable study findings of Confucian culture’s role in modern business management and cross-cultural conflict management in the academic field both abroad and at home. However, so far, the studies concerning the feasibility of integrating Confucian culture in the cross-cultural conflict management of Chinese firms going global along “BRI” are still rare in academia. With these significant research findings as to the foundation, this study combines the Confucian culture with Hofstede’s cultural dimension and further designs the questionnaires to conduct the empirical study.

## Methodology and Data

The study adopts core Confucian culture values of “He,” “Ren,” “Li,” and “Zhongyong” as four independent variables. It takes Hofstede’s four culture dimensions of power distance, uncertainty, individualism vs. collectivism, and masculinity vs. femininity as four dependent variables, thus creating the theoretical analysis framework. The questionnaire design is based on the integration of Confucian culture into cross-culture conflict management as dependent variables. The questionnaire survey findings aim to examine the feasibility of integrating Confucian culture into handling the cross-cultural conflicts faced by Chinese enterprises going global along “BRI.”

The employees of China’s multinational corporations who have already got involved with multinational business operations along “BRI” are invited to participate in the questionnaire survey. These employees are from diversified positions, including first-line workers, line managers, general managers, directors and even the board members who have different nationalities and diverse national cultural backgrounds. These enterprises are large and well-established in each industry, mainly Huawei Technologies, Guangdong Import and Export, and Zhongxing Telecom Equipment.

The questionnaire is designed according to the integration of Tsai et al. (2011) four core Confucian values with Hofstede’s four culture dimensions to fit the assumption of the specialty of research objects. The questionnaire consists of four parts. Part one is about demographic information collected to describe the survey participants’ individual information, which helps investigate the potential influence of unique features on Confucian culture resolution to cross-cultural conflict management. The second part is about cross-cultural conflicts of business management, designed to figure out the forms, origins and others of cross-cultural conflicts, thus paving a foundation for the following parts. Part three is about the Confucian culture, examining the participants’ cognition of Confucian culture and their assumption about the feasibility of integrating Confucian culture into cross-cultural conflict management. The final part is about the specific values of Confucian culture and whether the integration into cross-culture conflict management is effective or not. The questionnaire compilation fully takes the core Confucian culture values, Hofstede’s four culture dimensions and diversity of cultural background mentioned above.

Before the full questionnaire survey, the pilot study is done by selecting 12 participants. We aim to determine the deviations in the questionnaire that do not accord with the actual situation and proofread them. Besides, the questions’ wordings and orderings are adjusted to improve the quality of the questionnaire largely.

For convenience, the questionnaire survey is conducted through the online questionnaire APP, mainly by Wechat and email. The questionnaire APP is user-friendly, and the deadline is set to collect the well-done questionnaires from the participants. Because of the automatic recording of IP addresses, repetitions and invalid answers can be properly prevented. The survey finally collects 293 questionnaires from the participants, among which 264 are valid ones, with the ratio of collection reaching up to 90.1%. The left 29 questionnaires are invalid due to these reasons, incompleteness or the failure to open the documents. The following data analysis is done on 264 valid questionnaires.

## Findings

### Reliability and Validity Tests

Reliability reflects the consistency and steadiness of the result of the test tool. It is the indicator of the tested samples’ authentic characteristics. This paper mainly adopts the method of Cronbach α index with its value range between 0 and 1. When the α index is below 0.6, it is assumed that the validity is insufficient. If so, the questionnaire should be adjusted and improved. However, if the α index value is more than 0.6, it proves that the validity is of good effect. This study’s question samples are tested with this method, and the test results show that Cronbach α indexes are all more than 0.80, and a majority of them are more than 0.90, which shows that this survey’s result is very reliable. The detailed test results are seen as follows in Table 1.

**TABLE 1:** Reliability results of confucian cultural values.

Index (items: 4)	Cronbach’s α value	
“He”	0.934	
“Ren”	0.927	
“Li”	0.920	
“Zhongyong”	0.898	
Samling Kaiser-Meyer-Olkin Measurement	0.957	
Bartlett’s Degree of Sphericity	Chi-square Stat.	467.105
Degree of Freedom	120	
Significance	0.000	

Validity is the tool for testing the accuracy of the result. The validity test mainly aims to justify the structural validity of the testing tool. Before the factor test is done, the research first uses KMO and Bartlett’s statistical method to test each variable’s coefficient. It is generally considered to be the better effect when the KMO value is closer to 1. It is in a very good effect when the value is more than 0.9. However, it is assumed to be in a poor effect when the value is less than 0.6. Furthermore, the validity test shows that the possibility of Bartlett’s degree of sphericity is 0.000, less than 1%, indicating the correlation is very strong and available for factor analysis.

KMO’s measured value is 0.957, which justifies that the factor analysis result is consistent. The Chi-square value of Bartlett’s degree of sphericity in the measuring scale is 4,607.105, indicating that the research’s questionnaire data is of high correlation.

### Demographic Variables

The first part of the questionnaire survey is about the participants’ demographic data consisting of gender, nationality, position, working time and company size. This data is very helpful to make clear the exact information of the surveyed participants. The detailed information is shown in Table 2.

**TABLE 2:** Demographic information.

Demographic variable	Type	Number	Frequency
Gender	Male	182	68.94%
	Female	82	31.06%
Nationality	Chinese	216	81.82%
	Foreign	48	18.18%
Position	First line workers	99	37.50%
	Line managers	100	37.88%
	Directors or above	65	24.62%
Working time	<1 year	117	44.32%
	1–3 years	75	28.41%
	3–5 years	50	18.94%
	>5 years	22	8.33%
Company size	<50 people	56	21.21%
	50–200 people	92	34.85%
	200–500 people	51	19.32%
	>500 people	65	24.62%

**The data is counted following the 264 valid questionnaires.**

We observe that the surveyed participants are more males than females in gender, among whom there are more Chinese than foreigners in nationality. The ratio of male vs. female is reasonable in multinational companies as international business requires more time and energy devotion while working in foreign countries. On this, the males have the advantages over the females. These surveyed companies are Chinese companies that are expanding the overseas market along “BRI.” Although there are foreign employees in them, the majority of employees could be Chinese reasonably. As for their position, different levels’ employees, including first-line workers, line managers and directors or above, took part in the questionnaire survey. Their working duration is from less than 1 year to at most 5 years. It is found that most of the participants have been working in these companies for less than 3 years. It is attributable to the specific industries in this survey. These companies are surveyed mainly in high-tech, trade or construction industries which highly requires updated expertise. Therefore, these companies annually recruit talented graduates from top Chinese universities to gain talents equipped with the updated information and cutting-edge technologies. Therefore, these workers are quite new in the companies. Moreover, the company size ranges from less than 50 people to more than 500 people, which is available for the study to examine the different sized companies’ cross-cultural conflict management strategies. “BRI” encourages all sized companies who have the relative capabilities to go global and contribute to the development of countries all over the world.

### The Cross-Cultural Conflicts in Multinational Enterprises

This part is designed to examine the forms and origins of cross-cultural conflicts in multinational companies. Table 3 shows the top raking forms and origins among the employees in these multinational companies. Furthermore, the activeness of management in resolving these cross-cultural conflicts is investigated too.

**TABLE 3:** Cross-cultural conflicts in multinational enterprises.

Item	Content	Number	Frequency
Form	Employees’ value deviation	203	76.89%
	Employees’ habit deviation	167	63.26%
	Managers’ leadership style deviation	152	57.58%
	Managers’ decision style deviation	97	36.74%
	Others	13	4.92%
Origin	Value	209	79.17%
	Language barriers	163	61.74%
	Body language	55	20.83%
	Work habit	178	67.42%
	Time concept	86	32.58%
	Others	3	1.14%
Activeness in resolving cross-cultural conflicts	Less active	75	28.41%
	Active	173	65.53%
	Very active	16	6.06%

The results show that there are major cross-culture forms in these companies. The top four forms are employees’ value deviation, habit deviation, managers’ leadership style deviation, and managers’ decision style deviation. In terms of cross-cultural origin, value is also the most influential factor. Meanwhile, language barriers, body language, and work habits largely lead to misunderstandings or daily work conflicts. Regarding management’s activeness in handling cross-cultural conflicts, 28.41% of employees are unaware of their management’s actions to deal with cross-cultural conflicts. However, 71.59% admit that the management is active or very active in resolving cross-cultural conflicts. This evidence implies that multinational enterprises attach much importance to cross-culture issues because they play a crucial role in performance success.

### The Feasibility of Confucian Cultural Values

With the survey of the first three parts done, it can be concluded that the major cross-cultural conflicts are in the forms of employees’ value deviation, employees’ habit deviation, managers’ leadership style deviation and decision style deviation etc. these conflicts are mainly attributable to the difference in value. Multinational enterprises are active in resolving these cross-cultural conflicts. On the assumption of the feasibility of integrating the Confucian culture, this study further explores what exact Confucian culture values can be taken to mitigate the cross-cultural conflicts (see Table 4).

**TABLE 4:** Feasibility of confucian cultural values.

Sub-values of confucian cultures	N	Minimum value	Maximum value	Mean	Standard deviation
H1.Harmony: valuing the harmony atmosphere of firms	264	1	6	4.39	1.235
H2.Diversity: respecting different cultures and traditions of people	264	1	6	4.56	1.216
H3.Union: making profits with united hearts	264	1	6	4.42	1.215
H4.Peace: resolving cultural conflicts in a peaceful and friendly way	264	1	6	4.57	1.216
R1.Humanism: Caring employees in a humanistic way	264	1	6	4.41	1.133
R2.Love: showing managers’ love to subordinates	264	1	6	4.47	1.123
R3.Equality: treating all in a way as you want to be treated	264	1	6	4.44	1.162
R4.Imitativeness: disciplining themselves with inner attitudes of activeness	264	1	6	4.42	1.171
L1.Politeness: treating diverse people in a polite way	264	1	6	4.67	1.054
L2.Rules-based: regulating people’s behaviors based on rules and norms	264	1	6	4.46	1.136
L3.Self-awareness: strictly requiring themselves by exemplarily obeying rules	264	1	6	4.51	1.075
L4.No-prejudice: treating people of cultural diversity with no prejudice	264	1	6	4.63	1.050
ZY1.Moderation: managing cross-cultural conflicts in a moderate or participative way	264	1	6	4.27	1.144
ZY2.Flexibility: making and adjusting decisions according to changes and situations or in an innovative way	264	1	6	4.58	1.107
ZY3.Action-orientation: managing cross-cultural conflicts by doing rather than by saying	264	1	6	4.48	1.163
ZY4.Charity: interacting all cultures with social responsibility and kindness	264	1	6	4.47	1.113

Based on the four core values of Confucian culture, “He,” “Ren,” “Li,” and “Zhongyong,” this study refers to The Analects. It lists 16 sub-values of Confucian culture, which may be adopted to manage multinational companies’ cross-cultural conflicts. The participants are asked to rate their feasibility ranging from the least feasibility (minimum value 1) to the most feasibility (maximum value 6). The results indicate these values are very feasible because their average mean is between 4.0 and 5.0, close to the maximum value of 6. There is no doubt that different values cause cross-culture conflicts. There is no possibility for the employees with different nationalities to unify their values. However, the survey implies that Confucian culture values can be integrated to the diversity of cultures and create a harmonious atmosphere in multinational enterprises, thus mitigating the conflicts and improving performance.

## Conclusion

This study adopts both quantitative and qualitative research methods such as the questionnaire survey, the literature review, and the Cronbach α index method to explore the feasibility of integrating Confucian culture into cross-cultural conflict management. The findings are as follows: Firstly, the survey finds out the major forms of cross-cultural conflicts popularly recognized by employees in Chinese multinational enterprises. They are specifically employees’ value deviation, habit deviation, managers’ leadership style deviation, and managers’ decision style deviation. And these cross-cultural conflicts’ primary origins are recognized as value, language barrier, body language, etc. Furthermore, most questionnaire survey participants confess that their management has already recognized the importance of cross-cultural conflicts and is active in dandling them. Secondly, the questionnaire further discovers that more than half of the participants agree that integrating Confucian culture into cross-culture conflict management is feasible. With the coverage of four core values of Confucian culture “He,” “Ren,” “Li,” and “Zhongyong,” the related 16 sub-values are tested to be feasible in cross-cultural conflict management.

These findings first shed light on the existing literature as this study explores cross-culture conflict management from a new perspective beyond traditional researches. Moreover, the findings greatly contribute to Chinese multinational enterprises going global along “BRI.” These enterprises should readjust their cross-culture conflict management by considering Confucian culture. The findings empirically show that Confucian culture can be integrated into diverse national cultures and is feasible in cross-culture conflict management.

Our findings imply that Chinese enterprises should consider new strategies to manage cross-culture conflicts, especially during the COVID-19 pandemic (Tang et al., 2021). First, the management of the enterprises values the importance of learning Confucian culture. Confucian culture should be treated as the root of the corporate culture. While going global along “BRI,” enterprises have to encounter the challenges of cross-cultural conflicts. With Confucian culture support, enterprises may find more efficient ways to mitigate these cross-cultural conflicts, which may back up their sustainable global business operations success (Pan et al., 2021). Therefore, Chinese multinational firms need to learn the Confucian culture and apply it in cross-culture conflict management. Second, our study also proposes that the management should strengthen the training of intercultural communication ability. The training should incorporate the host countries’ diverse cultures, the cultural shock encountered in the host countries, and the cross-cultural communication skills. This training will help multinational employees handle cultural diversity when exposed to a strange cultural environment. In doing so, multinational enterprises may have fewer cross-cultural conflicts while going global. Third, multinational enterprises should value the importance of diverse cultural elements. “BRI” proposes harmony without uniformity. Respecting different cultures and embracing diversity is the foundation of cross-cultural conflict management, which conforms to the values of “He,” “Ren,” “Li,” and “Zhongyong” in Confucian culture. Chinese multinational companies going global along “BRI” are encouraged to take the local cultures’ essence and actively integrate Confucian culture to resolve the cross-cultural conflicts.

The questionnaire data collected are all from Chinese multinational enterprises with international operations in foreign markets along “BRI.” The data may be limited in that the scale and coverage of samples are still insufficient. Major participants are Chinese, and it is suggested to take more foreigners with diverse nationalities as the participants when a similar study is conducted. Future studies can enhance our findings by considering these issues.

## Data Availability Statement

The raw data supporting the conclusions of this article will be made available by the authors, without undue reservation.

## Ethics Statement

The survey in this research has been approved by the Ethical Committee of Guangdong University of Foreign Studies. The patients/participants provided their written informed consent to participate in this study.

## Author Contributions

XL: writing original draft and data collection. WZ: writing original draft. YL: writing and reviewing original draft. All authors contributed to the article and approved the submitted version.

## Conflict of Interest

The authors declare that the research was conducted in the absence of any commercial or financial relationships that could be construed as a potential conflict of interest. The reviewer ZW declared a shared affiliation, with no collaboration, with one of the authors, XL, to the handling editor at the time of the review.
